# Deep Learning to Predict Falls in Older Adults Based on Daily-Life Trunk Accelerometry

**DOI:** 10.3390/s18051654

**Published:** 2018-05-22

**Authors:** Ahmed Nait Aicha, Gwenn Englebienne, Kimberley S. van Schooten, Mirjam Pijnappels, Ben Kröse

**Affiliations:** 1 Department of Computer Science, Amsterdam University of Applied Sciences, 1091 GM Amsterdam, The Netherlands; b.j.a.krose@hva.nl; 2 Human Media Interaction, University of Twente, 7522 NH Enschede, The Netherlands; englebienne@gmail.com; 3 Neuroscience Research Australia, University of New South Wales, Sydney 2031, Australia; k.vanschooten@neura.edu.au; 4 Department of Human Movement Sciences, Vrije Universiteit Amsterdam, 1081 HV Amsterdam, The Netherlands; m.pijnappels@vu.nl; 5 Informatics Institute, University of Amsterdam, 1098 XH Amsterdam, The Netherlands

**Keywords:** accidental falls, older adults, machine learning, neural networks, convolutional neural network, long short-term memory, accelerometry

## Abstract

Early detection of high fall risk is an essential component of fall prevention in older adults. Wearable sensors can provide valuable insight into daily-life activities; biomechanical features extracted from such inertial data have been shown to be of added value for the assessment of fall risk. Body-worn sensors such as accelerometers can provide valuable insight into fall risk. Currently, biomechanical features derived from accelerometer data are used for the assessment of fall risk. Here, we studied whether deep learning methods from machine learning are suited to automatically derive features from raw accelerometer data that assess fall risk. We used an existing dataset of 296 older adults. We compared the performance of three deep learning model architectures (convolutional neural network (CNN), long short-term memory (LSTM) and a combination of these two (ConvLSTM)) to each other and to a baseline model with biomechanical features on the same dataset. The results show that the deep learning models in a single-task learning mode are strong in recognition of identity of the subject, but that these models only slightly outperform the baseline method on fall risk assessment. When using multi-task learning, with gender and age as auxiliary tasks, deep learning models perform better. We also found that preprocessing of the data resulted in the best performance (AUC = 0.75). We conclude that deep learning models, and in particular multi-task learning, effectively assess fall risk on the basis of wearable sensor data.

## 1. Introduction

Falls among older adults are one of the major health problems that lead to a decreased quality of life and increased morbidity and mortality. In addition, falls pose high costs to the public health service. Risk factors for falls include weak muscles, unsteady gait, cognitive decline, and psychoactive medications. Early detection and monitoring of fall risk factors can significantly reduce the risk of future falls [1,2]. Among these factors, history of falls and of gait and balance disorders have been identified as strong predictors [3].

Fall risk assessment is a process in which the probability of a future fall is estimated, usually within a time frame of 6–12 months. In many intervention programs proposed for fall prevention, fall risk assessment is performed as the initial step to identify persons at highest risk. The assessment of fall risk is commonly conducted in a clinical setting and based on questionnaires and functional tests of mobility such as the Timed Up and Go (TUG) [4], the Performance Oriented Mobility Assessment (POMA) [5], or the Berg Balance Scale test [6]. Although these tests provide a good indication of one’s optimal mobility and performance, their predictive ability for prospective falls is limited (e.g., [7]), possibly because this optimal ability might not be representative of one’s use in daily life behavior.

In previous research, we studied the use of ambient sensors for the continuous monitoring of human activities in their natural environment [8,9]. In this paper, we focus on body-worn inertial sensors that are used in many research studies on the ambulatory monitoring of humans in daily life providing reliable insight into an individual’s daily activities and gait quality characteristics [10].

Much research is done in the characterization of the quantity of movement of subjects, including the duration of low-, moderate-, and high-intensity activities, the total number of daily steps, and the daily percentage of time spent lying, sitting, standing, and walking [10]. Recent research showed the added value of the characterization of one’s quality of movement in the determination of fall risk in older adults [11]. These studies revealed that biomechanical features such as gait stability, variability, and smoothness [12,13], but also mean turn duration [14] and the number of abnormal sit-to-stand transitions [15], are associated with fall risk. However, estimation of these features often requires event detection, which is in need of improvement, and may not currently exploit the wealth of information that has been collected. On the other hand, deep learning allows for the data-driven generation of features and does not suffer from these shortcomings.

In machine learning, deep convolutional and long short-term memory (LSTM) recurrent neural networks have shown to be successful for the recognition of activities [16] and gait patterns [17] from inertial sensor data. However, the assessment of fall risk with such models has not been done before. The contributions of this paper are (a) a comparison of the performance of deep learning models for the assessment of fall risk with a baseline model based on biomechanical features using a large data set of 296 subjects and (b) the extension and testing of these models with multi-task learning to improve their performance.

## 2. Sensor Data

The data used in this paper were collected between March 2011 and January 2014 as part of the fall risk assessment in older adults (FARAO) cohort study performed at the Vrije Universiteit Amsterdam. The FARAO study collected data on fall risk factors in older adults with questionnaires, physical tests, and wearable sensors. Participants in the cohort were between 65 and 99 years of age, had a mini mental state examination score (MMSE [18]) between 19 and 30, and were able to walk at least 20 m with the aid of an assistive device, if needed. We re-analyzed the data described in [21], which consisted of a population of 296 older adults. These participants wore a triaxial accelerometer (Dynaport MoveMonitor, McRoberts) on their lower back, which registered 3D trunk accelerations at 100 Hz and ±6 G, for 1 week. During a 6-month follow-up period in which fall incidences and descriptions were obtained monthly, 101 subjects (34.1%) had experienced at least one fall and were identified as fallers. Table 1 provides an overview of the descriptive characteristics of the population. A detailed description of the population and the methods for data collection can be found in [11,19,21].

**Figure 1:**
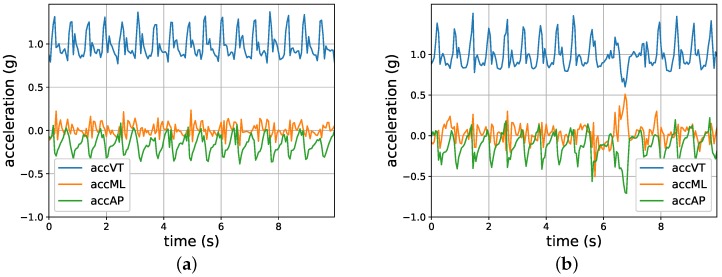
An example of two locomotion samples. (**a**) A typical walking sample; (**b**) A walking sample interwoven with a turning activity at 7 s. accAP: acceleration in the anteroposterior direction; accML: acceleration in the mediolateral direction; accVT: acceleration in the vertical direction.

Participants were instructed to wear the accelerometer with an elastic belt around their lower back at all times, except during aquatic activities such as showering. The distribution of the total time that the sensor was worn for fallers and non-fallers was similar. Bouts of non-wearing, locomotion, sitting, lying, and standing were identified using the manufacturer’s activity classification algorithm [20]. Only the locomotion bouts were analyzed in the current study. For each locomotion bout, the acceleration in three directions (i.e., anteroposterior (AP), mediolateral (ML), and vertical (VT)) was recorded. Figure 1 shows two examples of locomotion bouts lasting 10 s each.

## 3. Approach

On the basis of this data set containing bouts of accelerometer data from 296 participants and the identification of the participants into fall or non-fall categories, a model was made that predicts falls from accelerometer data. In van Schooten et al. [21], a linear model was used, based on biomechanical features from the accelerometer data. In this paper, we used deep neural networks. Deep learning allows for the creation of computational models that are composed of multiple processing layers and learn representations of data with multiple levels of abstraction [22]. This can result in more powerful models, because the complexity of the feature computations are dictated directly by the data and by the quality of the model predictions, rather than by the preconceptions of the operator. On the other hand, no prior knowledge is leveraged in the creation of the model, so it is useful to compare deep learning approaches to traditional machine learning methods.

We evaluated two types of deep neural network (DNN) for the analysis of fall risk. First, we considered the convolutional neural network (CNN), which constrains the number of parameters by sharing parameter values in different parts of the network. It has been used with great success in speech recognition [23] and in the detection, segmentation, and recognition of objects and regions in images [24,25]. We then looked at the long short-term memory (LSTM) model, a specific type of recurrent neural network (RNN). RNNs specifically model sequential inputs such as speech and language [26,27]. In this work, we used a model that combines convolutional and recurrent models, which we refer to as the “ConvLSTM”.

We trained the model parameters and evaluated the resulting models by minimizing the loss, a function that expresses how many prediction errors the model makes, and evaluated the models for different values of their so-called “hyper-parameters”, which include the number of layers and the number of nodes in each layer, based on their the receiver operating characteristic (ROC) curves. The models can make different types of errors, as well as false positive and false negative predictions, and a single model can be tweaked to minimize one type of error at the expense of the other. The ROC curve shows the model’s performance for multiple choices of this trade-off. The area under the ROC curve (AUC) is a robust metric of a model’s performance. The training, validation, and testing of the DNN was performed on a Distributed ASCI Supercomputer 5 (DAS-5) server [28].

## 4. Deep Learning Neural Network Models

### 4.1. Feed-Forward Neural Networks

**Figure 2:**
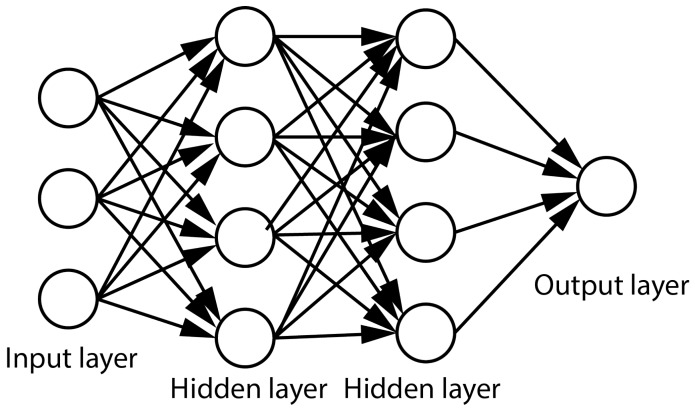
A three-layer neural network with three input neurons, two hidden layers of four neurons each, and one output layer.

Deep neural networks (DNNs) consist of large numbers of simple processing modules, the “neurons”, which compute a fixed function—the “activation function”—of the weighted sum of their inputs and are organized in separate layers. The simplicity of the neurons make network training possible, while the large number of nodes and their organization in a large number of layers allows them to perform complex tasks. DNNs have the ability to learn representations of the training data and relate them to the output variable(s) that we train them to predict. An example of a DNN consisting of two hidden layers is given in Figure 2. The number of nodes in the input layer is determined by the dimensionality of the data, while the number of nodes in the output layer is determined by the chosen representation of the intended prediction. The structure of the network is determined by the complexity of the task being predicted. In addition to the number of nodes and layers, the connections between layers affect the complexity of the network. In a dense layer, each neuron is connected to all neurons of the previous layer and has its own set of weights. In a convolutional layer, a neuron is connected to a subset of the neurons in the previous layer, and shares its weights with the other neurons of that layer.

### 4.2. Long Short-Term Memory (LSTM) Network

**Figure 3:**
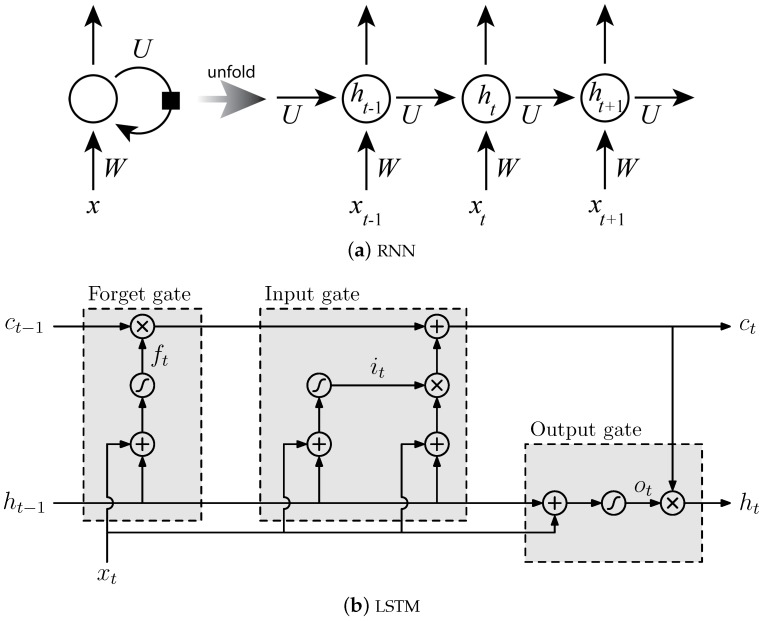
(**a**) A cyclic connection of a recurrent neural network (RNN) folded and unfolded. (**b**) An long short-term memory (LSTM) memory block consisting of one cell at time *t* and the three gates (*i_t_*, *o_t_*, and *f_t_*) which control the activation of the cell *c_t_* and its output *h_t_*.

Recurrent neural networks are a type of neural network where inputs are organized sequentially, and the output at time *t* is connected to all inputs from time 0 to *t* (Figure 3a). Such a network is still a feed-forward network, but the number of layers between an output and previous inputs increases as the time difference increases. In practice, the training of recurrent neural networks (RNNs) with long-term temporal dependencies can be problematic because the gradient of the loss function decays exponentially with the number of layers and, therefore, with time [29]. LSTM networks, introduced by Hochreiter and Schmidhuber [30], are a type of RNN that uses special units to solve this so-called vanishing gradient problem by “gating” the propagation of information over time. They extend RNNs with memory blocks (Figure 3b) to store information, easing the learning of temporal relationships over long time periods.

### 4.3. Multi-Task Learning

Multi-task learning (MTL) has been proposed by Caruana [31] to learn several related tasks by a single model. Having a network learn multiple tasks increases the complexity of the function it computes, but when the tasks are related, the models can share parameters. The complexity of a network performing multiple tasks is then lower than the complexity of multiple networks learning the tasks separately. In addition, the fact that tasks essentially compete for the resources of the network tends to force the network to avoid modeling non-essential aspects of the problem, thereby also improving the performance on the individual tasks.

## 5. Experiments and Results

We conducted a set of five experiments to evaluate the presented approach. In the first experiment, we compared deep neural networks (DNNs) with the current state-of-the-art model described in Section 5.1, which relies on manually engineered feature extraction. In the second experiment, we investigated the performance of DNNs in the prediction of fall status at the sample level (i.e., when allowing the model to train and test on different data from the same person), and show drastically improved results. In the third experiment, we explored whether these improvements are due to the model learning to identify people from their gait, rather than from better modeling of fall risk. We observed that the model is capable of identifying people from their gait, but that this does not by itself explain all of the performance increase. In the fourth experiment, we therefore explored how person-specific but not fall-related information can improve the model. We showed that multi-task learning improved fall prediction. Finally, in the fifth experiment, we showed how improving the focus of the model on cleaner data further improved the overall prediction performance. To train a model and calculate its performance, the complete dataset was split into a training and a validation set (90%) and a test set (10%).

### 5.1. Experiment 1

We compared the performance of three types of DNN using raw inputs to the performance of the state-of-the-art model. This base model was previously described by van Schooten et al. [21], and is based on a dataset of ten-second gait samples from which several features such as walking speed, variability, smoothness, and complexity were extracted. Principal components analysis (PCA) was applied to these features (as well as other parameters obtained from questionnaires and tests), keeping 18 principal components, and a multivariate model was developed to predict time to prospective falls. The median of a person’s ten-second segments’ predictions provided that person’s risk assessment. This base model resulted in a performance of AUC = 0.67 (95% confidence interval [0.59, 0.73]) at 6 months [21].

**Figure 4:**
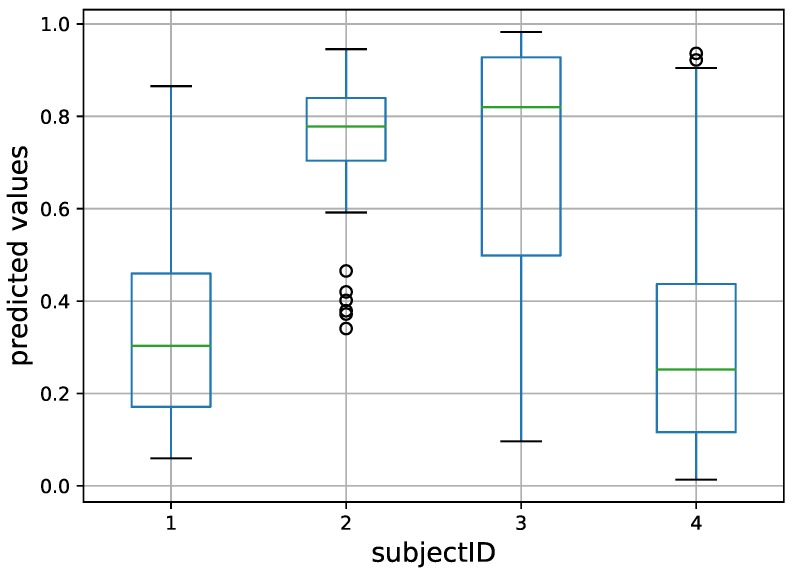
Example boxplots of the normalized predicted values ($\hat{y}$) for multiple 10-s sequences, grouped by subject. Subjects 1 and 4 were non-fallers and the other two were fallers. The final prediction per subject was given by the median of the predictions, as per van Schooten et al. [21]. The green line inside the box represents the median, the box represents the range of first and third quartile and circles represent outliers.

The same complete dataset was randomly split into three subsets (training, validation, and testing) at the subject level, where all the 10-s samples of a subject *A* occur in a single subset. The ratio of fallers to non-fallers was approximately the same in these three sets. The DNNs were given 10-s samples *x* and the corresponding faller/non-faller label y∈{0,1} for training and testing. For each sample *x*, the predicted value using a DNN architecture was denoted by $\hat{y}$. The median of the predicted values for all of a subject’s samples was used as the predicted value for that subject. The subjects’ predicted values and their actual values (label) were used to plot the ROC and to calculate the corresponding AUC. Figure 4 shows an illustration of predicted values ($\hat{y}$) for multiple 10-s sequences grouped by subject.

**Figure 5:**
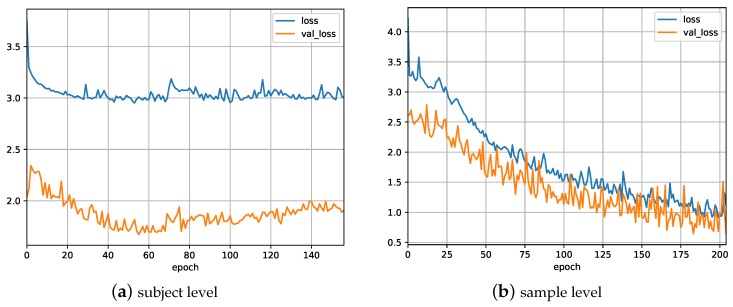
A typical loss versus epoch graph during the training of a deep neural network (DNN). The data has been split at (**a**) subject level or (**b**) sample level. Loss is the training loss and val_loss is the validation loss. The gap between the training and validation loss indicates the amount of over-fitting.

**Figure 6:**
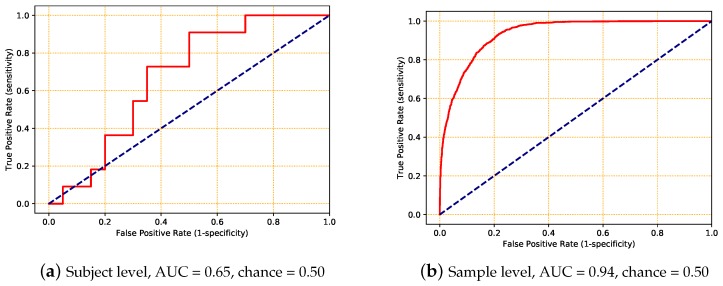
Examples of the receiver operating characteristic (ROC) curves (solid red lines) and their corresponding area under the curve (AUC) values obtained using a ConvLSTM model. The dashed blue line represents the ROC for chance. The dataset was split at (**a**) the subject level and (**b**) the sample level.

**Figure 7:**
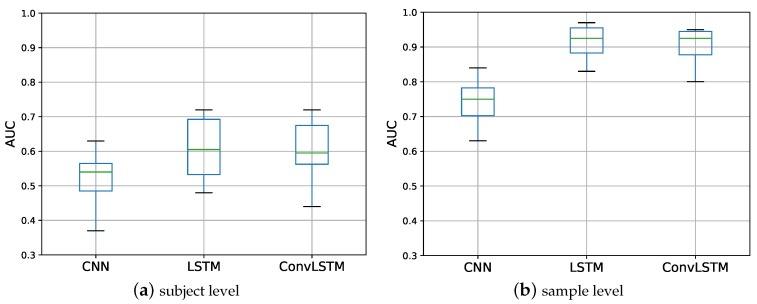
A boxplot of the AUCs of different DNN architectures. For the LSTM architecture, at least two LSTM layers were involved, while for the ConvLSTM architecture, only one LSTM layer was involved. The dataset was split at (**a**) the subject level and (**b**) the sample level.

As described in Section 3, three types of DNN architectures (CNN, LSTM, and ConvLSTM) were applied to a small set of the data to determine the best-fitting model. The models were trained by minimizing the binary cross-entropy loss function (Figure 5a), and evaluated in terms of the area under the ROC curve for each subject (Figure 6a). The corresponding AUC was used to measure the performance of the models (Figure 7a).

From these, we can conclude that the LSTM and ConvLSTM architectures resulted in a slightly better performance than the CNN architecture (*p*-values were, respectively, 0.056 and 0.022) and that there was no significant difference in the performance between LSTM and ConvLSTM (*p* = 0.480). The time needed for the training of the LSTMs was very long compared to the ConvLSTM architecture (Table 2), because two or more LSTM layers were used in the LSTM architecture while the ConvLSTM architecture was set to have exactly one LSTM layer. For this reason, we selected a ConvLSTM architecture and its corresponding hyper parameters to be trained on larger datasets. Table 3 illustrates the architecture of the ConvLSTM type used. The AUC and the corresponding training time of this architecture are given in Table 4.

We compared the performance, in terms of average AUC, of the best-fitting model to the base model using a *z*-test, and found no statistically significant difference (*p* = 0.209). In addition, the results also showed a poor generalization ability of the DNN model when trained at the subject level, as indicated by the gap between the two loss functions in Figure 5a. Perhaps the model learned concepts from the training data that did not apply to the test data and therefore negatively impacted the performance of the model. For the investigation of the cause of this generalization problem, we conducted a second experiment, where we applied the same types of DNNs on different training and testing subsets.

### 5.2. Experiment 2

For this experiment, the complete dataset was randomly split into three subsets (training, validation, and testing) again, but now at the sample level. As a consequence, there was only a small chance that all of the 10-s samples of a single subject were allocated to only one subset. As in the first experiment, we tested three DNN architectures on a small set of the data to identify the best-performing architecture. The ConvLSTM architecture again resulted in the best trade-off between performance and training time (Figure 6b and Figure 7b). A *t*-test showed that both LSTM and ConvLSTM had a significantly better performance than CNN (*p* < 0.002), and there was no significant difference between LSTM and ConvLSTM (0.580). Furthermore, this experiment resulted in a better performance than the previous experiment, as shown in Table 5.

The high AUC when splitting the data at the sample level compared to the subject level can be explained by the smaller within-subject, compared to between-subject, variability of gait. However, another explanation may be that the model learns to identify subjects better than it recognizes characteristics indicating fall risk (since the same subjects were present in training and testing sets, the model could map their identity to fall risk). In the third experiment, we checked the model’s ability to identify subjects’ gait signatures.

### 5.3. Experiment 3

**Figure 8:**
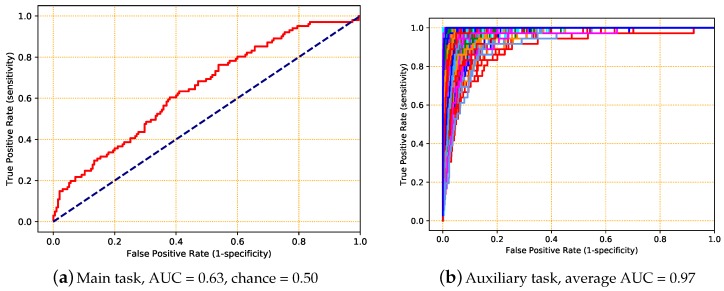

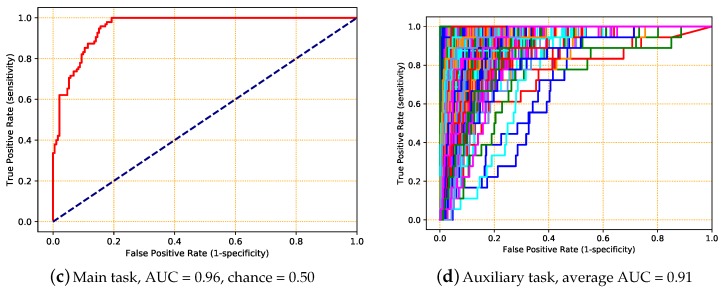
A sample of obtained ROCs for multi-task deep learning (MTDL) with fall status as the main task and subject identity as the auxiliary task. For the auxiliary task, the ROCs were computed using one-versus-all. The corresponding average AUC is reported. For (**a**,**b**), the main and auxiliary losses were given the same weight (1:1); for (**c**,**d**), the main loss function was given higher weight (10^4^:1) than the auxiliary loss function. The dashed blue lines in (**a**,**c**) represent the chance ROC.

We again split the dataset into three subsets at the sample level. To learn subject signatures together with their fall risk, we used multi-task learning (MTL): fall risk was the main task, while the identity of the subject was the auxiliary task. We used the same ConvLSTM architecture as in Table 3, because of its good trade-off between performance and learning time in the previous experiments, with an additional dense output layer (connected to Layer 11) for the auxiliary task. The overall loss of the network is a weighted sum of the losses on the main and auxiliary tasks. Ten-fold cross-validation was used to calculate the performance of both the main and auxiliary tasks. The performance of the auxiliary task, which identified the person out of the 296 in the dataset, was evaluated with a plot of the ROC for each subject in a one-versus-all approach. The ROC of the main task and, for clarity, a random sample of the ROCs for the auxiliary task are shown in Figure 8.

As we can see, when both tasks were given the same weight (Figure 8a,b), the network was exceedingly good at recognizing identities, but not as good at predicting fall risk. The network had the information to learn the mapping from identity to risk, but not the information capacity to learn this mapping. Therefore, when we increase the weight of the main task, the network becomes better at predicting fall risk at the expense of the identification task (Figure 8c,d). From this, we could conclude that there were important differences between subjects, but that there were other informative patterns in the data, which led us to our next experiment.

### 5.4. Experiment 4

In this experiment, we investigated the effect of MTDL on the model performance. When we split the data at the subject level, it makes no sense to use subject ID as the auxiliary task (since the IDs in the test set are never seen during training), but other subject characteristics can form an informative auxiliary task. The experimental setup is similar to the previous experiment, except that the data was split at the subject level and the auxiliary task was one of the following subject characteristics: *age*, *gender*, *weight*, and *height*. Table 6 shows the average AUC and the corresponding standard deviation of the main task (fall status). We can conclude that MTDL consistently resulted in improved performance compared to the single-task learning used in the first experiment. However, the improvement was not significant when compared to the base model.

### 5.5. Experiment 5

**Figure 9:**
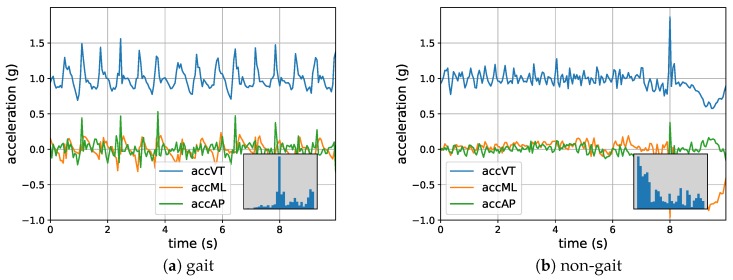
(**a**) An example of a 10-s data sample included in the training and testing set and (**b**) an example of a data sample excluded in Experiment 5 due to the low dominant frequencies in the VT-axis. In the bottom-right corners, histograms of the VT-frequencies up to 3 Hz are depicted. Both examples were included in the first 4 experiments.

In the previous experiments, we used the exact same 10-s data segments as found in van Schooten et al. [21], which consist of samples of locomotion as identified by the accelerometer manufacturer’s algorithm [20]. As the data were collected in a daily living environment, the locomotion bouts may contain some “non-gait” data samples, which may have negatively affected the performance of the DNNs. Visual inspection of the data indeed suggested the presence of such data samples. These data samples correspond to cyclic accelerations of the trunk without taking clear steps (e.g., when riding a bike) or involve only a few steps (e.g., when moving in the kitchen while preparing a meal). The objective of this experiment was to investigate the effect of conservatively selecting gait data samples on the performance of the models. To do so, 10-s data samples having a very low dominant frequency in the vertical direction (VT-axis) (≤0.2 Hz) were removed from the data, resulting in approximately 20% discarded data. An example of such included and excluded samples is shown in Figure 9. A procedure similar to Experiment 4 was followed to train, test, and calculate the performance of the ConvLSTM model. Table 6 shows the obtained average AUC and the corresponding standard deviation of the main task. It should be noted that, although different data were used for both training and testing, the results are per *subject*. Therefore, for the same subjects, they are comparable. Comparing these results with those of Experiment 4, we may conclude that the excluded samples did have a negative effect on the performance of the DNNs. The obtained results of the *z*-test showed that this model resulted in a significant improvement compared to the performance of the base model. These results suggest that improvement in fall prediction based on accelerometry is not only warranted on the modeling side, but also on the input (or activity classification) side.

## 6. Discussion and Conclusions

In this paper, we studied the use of deep learning neural networks to model fall risk on the basis of accelerometer data. Our aim was to compare the performance of deep learning on raw acceleration data with the performance of a base model that uses biomechanical features extracted from the data. For this comparison, we used the same dataset. We did not compare our approach with other work done on different tasks such as activity recognition [16] or age-related differences in walking [17].

In our first experiment, we selected the ConvLSTM neural model based on its trade-off between performance and training time. However, although we found that this architecture was best in modeling the training data, it generalized poorly over subjects. This was confirmed in Experiment 2, where we achieved a very good performance (AUC = 0.94) when the training and validation sets contained data, split at samples, from all 296 subjects. The very good performance in this case may have been caused by the network learning identities of subjects from gait data and using these implicitly to model individual fall risk. In Experiment 3, we studied an MTL network that simultaneously modeled fall risk and identity. We inferred the need to control for subject-specific factors, since training for both fall risk and identity improved the model’s performance considerably. In Experiment 4, we studied an MTL approach where as auxiliary task we chose more general characteristics such as age or weight as secondary tasks that were still related to the subject, but were not the subject itself. We found an improvement of the performance of the ConvLSTM model on the validation set of new subjects if we used gender and age as auxiliary outputs. When we compared the performance of our MTL ConvLSTM with the base model of van Schooten et al. [11], we saw a slightly higher performance. However, this was not significant. Nevertheless, our results indicate that deep learning methods provide similar high accuracy of fall risk prediction compared to biomechanical models, with the advantage that they do not require painstakingly crafted features.

The performance of a model relies on the model architecture used and on the input data. In Experiment 5, we therefore studied an approach where we selectively ignored some of the data samples based on a spectral analysis. We found a significantly better performance. These results suggest that a stricter gait classification algorithm may result in more accurate identification of an individual’s gait signature and therefore improve model performance. Another option is to use the dynamics in the data over periods longer than 10 s. This can be done by using the entire locomotion bout as input for the ConvLSTM network. Another method is adopting hierarchical methods [32].

In conclusion, this work shows that machine learning on accelerometer data acquired in the home environment provides comparable accuracy to conventional models in the assessment of fall risk of older adults, with the advantage that they do not rely on handcrafted extracted features. We believe that this approach will contribute to the societal challenge of healthy and active aging in the home environment.

## Figures and Tables

**Table 1:** Descriptive statistics of the population.

	Male (%)	Age (Years)	Weight (kg)	Height (cm)
Mean	74.1	75.3	49.2	170.6
Standard deviation	-	6.8	13.3	8.8
25% Quantile	-	70.0	64.0	165.0
75% Quantile	-	80.0	81.8	176.0

**Table 2:** Average AUC (standard deviation) and corresponding average training time per neural network (NN) architecture type for a subset of the data. The difference in training time between the ways of splitting the data is due to the slower convergence when splitting at the sample level.

	Subject Level		Sample Level	
	AUC	Time (h)	AUC	Time (h)
CNN	0.52 (0.07)	6	0.74 (0.07)	7
LSTM	0.61 (0.10)	160	0.91 (0.06)	180
ConvLSTM	0.60 (0.09)	35	0.90 (0.05)	40

**Table 3:** The ConvLSTM architecture (ConvLSTM is our proposed model that combines convolutional and recurrent models). To keep the architecture clear, we omitted the input layer (layer 00) and the dropout layers (the even layer indices) applied after each convolutional neural network (CNN) layer. *N* was set to 128.

Layer Index	01	03	05	07	09	11	12
type of filter	CNN	CNN	CNN	CNN	CNN	LSTM	Dense
number of filters	*N*	*N*	*N*	$\frac{3}{4}N$	$\frac{3}{4}N$	*N*	2

**Table 4:** Average AUC, training duration, and number of folds obtained when applying the ConvLSTM model to different dataset sizes. The dataset was cut into three subsets at the subject level.

	Dataset Size in Minutes				
	10	30	60	120	Complete Dataset
Average AUC	0.61	0.63	0.65	0.65	0.65
Training duration (h)	35	90	150	250	350
Number of folds	10	10	10	2	1

**Table 5:** Average AUC and the corresponding standard deviation when splitting at sample or subject levels.

	AUC	
	Average	Standard Deviation
subject level	0.65	0.09
sample level	0.94	0.07

**Table 6:** Average AUC and the corresponding standard deviation of the main task (fall status), obtained when the ConvLSTM is applied to the test set. The *p*-value was obtained using the *z*-test to test the difference in the performance to the base model.

Characteristic	AUC Main Task (std dev)		*p*-Value Diff to Base Model	
	Experiment 4	Experiment 5	Experiment 4	Experiment 5
Gender	0.70 (0.06)	0.75 (0.05)	0.070	<0.001
Age	0.70 (0.05)	0.74 (0.05)	0.082	<0.001
Weight	0.68 (0.05)	0.72 (0.05)	0.306	0.005
Height	0.63 (0.06)	0.65 (0.06)	0.987	0.897
